# Neuromodulatory effects of high-definition theta transcranial alternating current stimulation on the parietal cortex: a pilot study of healthy males

**DOI:** 10.3389/fnins.2023.1255124

**Published:** 2023-11-09

**Authors:** Xixi Chen, Yuwei Wu, Xiaolong Shi, Zhiqing Zhou, Tingyi Feng, Meng Ren, Yuanli Li, Chunlei Shan

**Affiliations:** ^1^School of Rehabilitation Science, Shanghai University of Traditional Chinese Medicine, Shanghai, China; ^2^Institute of Rehabilitation, Shanghai Jiao Tong University School of Medicine, Shanghai, China; ^3^Department of Rehabilitation Medicine, Yueyang Hospital of Integrated Traditional Chinese and Western Medicine, Shanghai University of Traditional Chinese Medicine, Shanghai, China; ^4^Engineering Research Center of Traditional Chinese Medicine Intelligent Rehabilitation, Ministry of Education, Shanghai, China; ^5^Department of Rehabilitation, Shanghai Seventh People’s Hospital, Shanghai University of Traditional Chinese Medicine, Shanghai, China; ^6^Department of Rehabilitation Medicine, Tong Ren Hospital, Shanghai Jiao Tong University School of Medicine, Shanghai, China

**Keywords:** transcranial alternating current stimulation, parietal cortex, short latency afferent inhibition, EEG, nonlinear dynamics analysis

## Abstract

**Introduction:**

Transcranial alternating current stimulation (tACS) can regulate brain functions by modulating endogenous brain rhythms. Theta-band neural oscillations are associated with memory function. In particular, theta neural oscillatory power evoked in the parietal cortex is closely related to memory retrieval processes. In this study, the immediate effects of high-definition theta transcranial alternating current stimulation (HDθ-tACS) on the human left parietal cortex were investigated using short-latency afferent inhibition (SAI) and electroencephalography (EEG).

**Methods:**

Ten subjects participated in this study. We used 6-Hz HD tACS to stimulate the left parietal cortex for 15 min. SAI was calculated, and non-linear dynamic analysis of the EEG was performed to analyze neuronal function after HD θ-tACS.

**Results:**

The results showed a significant decrease in SAI (*p* < 0.05), while the left frontoparietal network was reinforced, leading to brain lateralization after HD θ-tACS. During performance of a memory task, F3 signals showed a significant upward trend in approximate entropy following treatment (*p* < 0.05). There was also a significant decrease in cross-approximate entropy in the C3–C4 and P3–P4 connections following the intervention (*p* < 0.05) in a resting eyes-open condition and in the memory task condition.

**Discussion:**

In conclusion, HD θ-tACS could alter cholinergic transmission and cortical excitability between the parietal and motor cortices, as well as reinforcing the frontoparietal network and the left-lateralization phenomenon, which may facilitate memory formation, encoding, and consolidation.

## 1. Introduction

Transcranial alternating current stimulation (tACS) is a non-invasive neuromodulation technique that can modulate neural oscillatory activity by means of sinusoidal oscillatory currents (Klink et al., 2020). High-definition (HD) tACS, a more focal type of tACS, employs a stimulus pattern with a central electrode surrounded by four return electrodes, which can contribute to a more focal electrical field distribution (Villamar et al., 2013). This focal distribution enables HD tACS to focus more precisely on the target and reduces its impact on other areas. Theta oscillations (3–8 Hz) are associated with short-term memory (Vertes, 2005), and stimulation in the theta band can induce or entrain theta oscillations in the brain. It has been shown that theta-tACS (θ-tACS) can increase short-term memory capacity (Vosskuhl et al., 2015), affecting performance in working memory tasks as well as P3 amplitude and latency (Pahor and Jaušovec, 2017). P3 is thought to be related to conscious information processing and is generally divided into two subcomponents: P3a is related to stimulus-driven attention processes in task processing, while P3b is related to attention and subsequent memory processing (Polich, 2007). Although several brain regions have been demonstrated to be associated with P3 sources, most sites of P3 oscillations are found in the parietal lobe (Leue and Beauducel, 2019; Li et al., 2021). In addition, some studies have found that the increase in theta-band power in neural oscillation that occurs during recognition is related to the process of making memory decisions, and that parietal evoked theta power is closely related to the memory extraction process (Wynn et al., 2019).

In previous studies, the parietal lobe has been shown to be associated with several brain functions, including cognitive control, memory retrieval, and short-term memory (Sheremata et al., 2018; Lee et al., 2019; Ray et al., 2020). In particular, it is involved in human memory processing. During memory retrieval, parietal reactivation induced by stimulation with different categories of items can predict memory outcomes (Gilmore et al., 2015; Lee et al., 2019). The left posterior parietal cortex (PPC) can independently encode familiarity in memory (Rutishauser et al., 2018). In addition, the PPC has been observed to exhibit elevated activation during extraction of learned episodic memories (Elman et al., 2013). Previous studies have shown that the damaged brain regions in patients with short-term memory impairment include the inferior superior gyrus and the angular gyrus, located in the left inferior parietal cortex (Vallar and Papagno, 1995). The relationship between the parietal lobe and short-term memory has been confirmed by analysis using electrophysiological imaging techniques and a hemispheric encoding/retrieval asymmetry model (Tulving et al., 1994; Constantinidis and Steinmetz, 1996; McCarthy et al., 1997). The abovementioned studies suggest that parietal regions, especially the left parietal lobe, are important for memory. Therefore, in this study, we used HD θ-tACS to stimulate the left parietal cortex to explore the effect of this intervention on memory abilities in healthy subjects.

Electrophysiological indices, such as short-latency afferent inhibition (SAI) and electroencephalography (EEG), can reflect changes in brain function and neuroexcitability to enable better understanding of the neuromodulatory effects of tACS. SAI is a special assessment modality used in transcranial magnetic stimulation (TMS), in which a peripheral nerve (such as the median nerve) is electrically stimulated before TMS. In this modality, adjusting the time interval between the two forms of stimulation (N20 latency +2–8 ms) can cause a decrease in cortical excitability. It has been demonstrated that SAI has high sensitivity in detecting the function of cholinergic circuits, and cholinergic activity in the cerebral cortex is related to cognitive function, such as memory (Di Lazzaro et al., 2000, 2005a). θ-tACS acting on the parietal cortex can affect memory, for instance, by increasing the storage capacity of working memory (Jaušovec et al., 2014; Jaušovec and Jaušovec, 2014). A previous study recorded the effects of SAI in participants during the encoding, consolidation, and retrieval phases of a recognition memory task. In that study, SAI was found to increase during retrieval, reflecting real-time activation of cholinergic transmission during the retrieval phase (Bonnì et al., 2017). It has been demonstrated that acetylcholine transmission changes during memory processes; however, it is unclear whether HD θ-tACS of the memory-related left parietal cortex is associated with the acetylcholine circuit and the neurotransmitter acetylcholine.

EEG, or the event-related potential (ERP), is commonly used to evaluate neural activity and its associated cognitive diseases (Donoghue and Voytek, 2022). Compared with linear analysis of EEG/ERP data, non-linear dynamic analysis (NDA), which uses a simple dynamic methodology to describe complex neural networks, can be used to quantify the complexity of EEG, provide information about the functions and interconnections of neural networks, and determine the trajectory of changes in brain functional activity, which cannot be obtained by conventional EEG analysis alone (Micheloyannis et al., 1998). For example, approximate entropy (ApEn) can be used to describe the complexity and regularity of a single brain region and can indicate the activity of that brain region, while cross-ApEn (C-ApEn) can help in judging the interconnection between two brain regions. Previous studies of attention and cognition have suggested that early Alzheimer’s disease (AD) patients can be identified based on the correlation dimension (D2) and non-linear prediction (Jelles et al., 1999). ApEn can also be used to explore the degree of altered cortical information processing and neurological dysfunction in children with attention deficit hyperactivity disorder (Sohn et al., 2010). Among previous studies related to cognition, non-linear EEG analysis has been applied mainly in studies related to schizophrenia and attention (Jelles et al., 1999; Sohn et al., 2010; Carlino et al., 2012), and only in a small number of studies related to memory. Like conventional EEG analysis, NDA has great potential value in memory-related studies.

In this study, we conducted HD θ-tACS to modulate the left parietal cortex in healthy subjects in order to observe the immediate SAI and to conduct NDA of the EEG responses. We hypothesized that HD θ-tACS would cause an increase in cholinergic circuit transmission and in activation of the parietal cortex. To test this hypothesis, we analyzed changes in cortical excitability before and after application of HD θ-tACS to observe the effects on cholinergic circuits. Additionally, we examined the function and connections of the neural network both pre- and post-stimulation. The findings are expected to enhance our understanding of the neural regulation mechanism of tACS.

## 2. Methods

### 2.1. Participants

Ten healthy male individuals (mean age: 24.6 years; range 21–35 years) (Smith et al., 2002) participated in this study. Nine individuals were right-handed and one was left-handed, as determined by the Edinburgh Handedness Inventory. Participants were excluded if they had local skin infections, ulcers, scarring, excessive sensitivity to electrical stimulation, a history of epilepsy, metal implants in the head, or other diseases or medical history that could affect cognitive function. Ethical approval was granted by Yueyang Hospital of Integrated Traditional Chinese and Western Medicine, Shanghai, China. The study was registered with the Chinese Clinical Trial Registry (ChiCTR2100052781).^1^ All participants provided written informed consent.

### 2.2. Procedure

Participants were instructed to sit comfortably in a chair. For each participant, the N20 latency and resting motor threshold (RMT) were recorded. Before and after the HD θ-tACS intervention, we assessed the mean amplitude of an unconditioned motor evoked potential (MEP) (without conditioning electrical stimulation) and a conditioned MEP (with conditioning electrical stimulation), as well as recording EEG signals during the memory task. Further, the SAI was calculated analytically. The memory task involved presenting a string of numbers to the participant and asking them to remember and repeat this. A number was added to the string each time it was presented.

### 2.3. Stimulation

The Soterix MxN TES device (Soterix Medical Inc., New York, USA) was used for stimulation. Before stimulation, we degreased the electrode placement area on the scalp with a scrub (Nuprep Skin Prep Gel; Weaver and Company, Aurora, CO, USA) and optimized the conductivity using a conductive paste (GT10; Greentek, Wuhan, China), while the impedance of each electrode was kept below 10 kΩ. According to the International 10–20 system for EEG, electrodes were placed at P3, CP3, P5, P1, and PO3, with P3 as the central electrode. The parameter settings for each HD θ-tACS session were as follows: a 30-s ramp-up and ramp-down time; 15 min of θ-tACS at 6 Hz and 0.75 mA (peak to baseline). The participants were at rest during the application of θ-tACS. Participants were asked whether they thought they had received a real stimulus at the end of each experiment and whether they had felt skin tingling. During the intervention, participants were monitored to avoid discomfort. For safety reasons and to avoid adverse consequences, we set the intensity to 0.75 mA.

### 2.4. SAI

Prior to the SAI protocol, we recorded somatosensory evoked potentials by electrically stimulating the right median nerve at the wrist in order to obtain the individual N20 latency for each participant. According to the International Standard 10–20 system, the recording electrode was placed at C′3 (3 cm behind C3) and the reference electrode was placed at Fpz. Stimulation electrodes were placed 2–3 cm above the medial transverse wrist and stimulated with square wave pulses at an intensity slightly above the motor threshold to induce a stable twitch of the thenar muscles. Two hundred responses were averaged to obtain the N20 latency.

Transcranial magnetic stimulation (TMS) was performed using MagVenture (MagPro R30, Denmark) with a figure-of-eight coil for intervention and localization. Electromyogram signals were acquired using Ag/AgCl surface electrodes (Kendall Medi-Trace), with the recording electrode placed at the muscle belly of the right first dorsal interosseus (FDI) muscle and the reference electrode at the FDI tendon. The ground electrode was placed at the forearm. The skin was degreased by wiping with a 95% alcohol cotton ball before application of the electrodes. The participants were asked to sit quietly in a chair with the right upper limb relaxed, and the coil was held over the left primary motor cortex (M1) at the optimum scalp position, with the coil placed tangential to the scalp and at 45° to the mid-sagittal line of the body. The optimal location of M1 was identified by initially determining the coil position according to the International 10–20 EEG positioning system. Next, the coil was moved in small increments within the hand region of M1 until the position was found where the maximum MEP was consistently obtained. The RMT is the minimum stimulation intensity at which more than 5 out of 10 consecutive trials generate amplitudes greater than 50 μV. TMS intensity and RMT values are presented as a percentage of the maximum stimulator output (% MSO). Conditioning electrical stimulations were delivered to the median nerve at the right wrist. In this study, the intensity of the conditioning stimulation (CS) was set to values that evoked a visible twitch of the thenar muscles, and the intensity of the test stimulation (TS) was adjusted to 120% of the RMT. In particular, the CS preceded TS, and the interstimulus interval between the CS and TS was the N20 latency +3 ms (Tokimura et al., 2000). In total, 10 unconditioned and conditioned MEPs with positive repeatability were recorded, and the average amplitudes were calculated for each individually. The SAI value was expressed as the ratio of the mean conditioned MEP to the unconditioned MEP (the smaller the SAI value, the stronger the inhibitory effect).

### 2.5. EEG recording

In this study, EEG signals were recorded using a wireless 16-channel digital EEG system (ZN7100, Zhineng Electronics, Chengdu, China). In total, EEG signals from 16 channels (FP1, FP2, F3, F4, C3, C4, P3, P4, O1, O2, F7, F8, T3, T4, T5, and T6) were recorded according to the international standard lead 10–20 system. EEG signals were acquired at a sampling rate of 500 Hz and bandwidth of 0.3–100 Hz, with a 12-bit analog-to-digital conversion resolution. The participants were initially seated comfortably with an EEG cap, and electrodes soaked in 0.9% saline were placed in the appropriate positions. EEG signals were sequentially recorded with participants in three states: quiet with eyes closed, quiet with eyes open, and performing the memory task. Data were acquired during the quiet-with-eyes-closed and quiet-with-eyes-open conditions for 5 min. Memory tasks were presented using the self-contained tasks available on the computer used for non-linear EEG analysis; in these tasks, a string of 3-10 non-consecutive numbers appeared on the screen. The starting phase began with 3 numbers and gradually increased the number of numbers tested, and the participants were required to memorize the numbers and repeat them after they disappeared. The entire task lasted approximately 3 min.

### 2.6. NDA of EEG

The ApEn and C-ApEn were calculated to analyze the EEG signals acquired in the three conditions.

#### 2.6.1. ApEn

ApEn was mainly used to describe the signal complexity and regularity. The higher the value, the higher the complexity of neuronal activity, and thus this index was used to monitor and directly measure the degree of cortical inhibition and excitability of the cerebral cortex during the task state in real time (Wu et al., 2011).

#### 2.6.2. C-ApEn

C-ApEn was used to measure the degree of dissimilarity between two synchronized brain regions, with higher values indicating more “microstates” between the two brain regions, indicating increased cortical interconnection or information flow (Wu et al., 2011).

### 2.7. Statistical analysis

Statistical analyses were performed using SPSS V.24.0. To examine changes in cortical excitability, the amplitudes of unconditioned MEPs before and after HD θ-tACS were compared using a Wilcoxon paired test. A paired *t*-test was conducted to assess the effect of HD θ-tACS on SAI, comparing pre- and post-intervention values. Non-linear EEG analysis was conducted using the original EEG signals, taking 30–45-s recording segments with a smooth waveform and few artifacts in each of the three states. The average value of each parameter for each lead was used for analysis. The measurement data were tested for normality of distribution. If continuous variables conformed to a normal distribution, they were compared using paired *t*-tests. If the measured values did not conform to a normal distribution, the non-parametric rank-sum test was used for comparison.

Normally distributed data are expressed in the form mean ± SD, whereas non-normally distributed data are presented in the form median (P25, P75). All data were checked using two-sided calibration with a test level of *α* = 0.05. *p* < 0.05 was taken to indicate a statistically significant difference.

## 3. Results

### 3.1. Effects of HD θ-tACS on SAI

**Figure 1:**
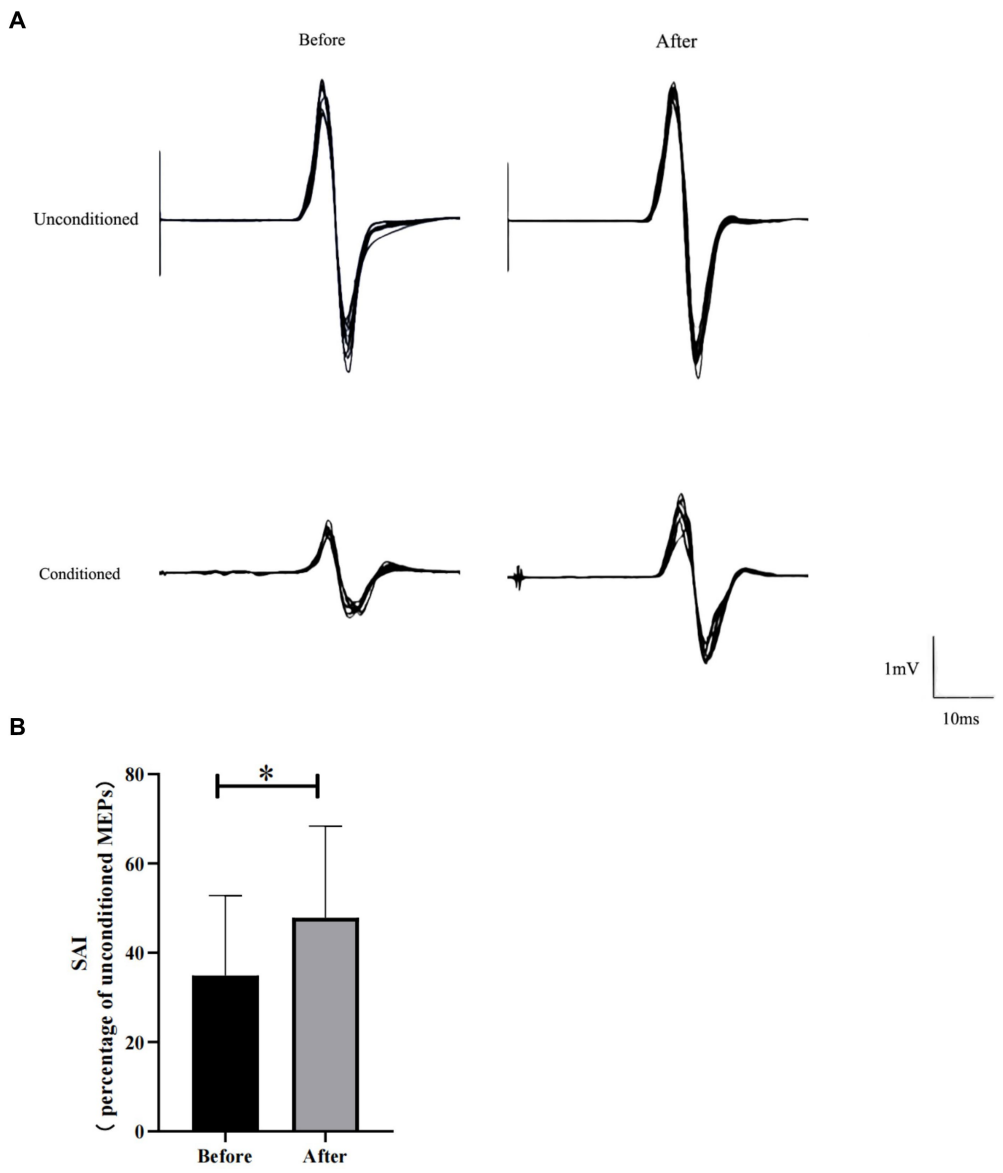
Effect of HD θ-tACS on SAI. **(A)** Each MEP waveform (black lines; *n* = 10) recorded from a representative participant before and after HD θ-tACS. **(B)** SAI before and after HD θ-tACS (*n* = 10). *Y*-axis: SAI expressed as % change relative to unconditioned MEPs (= 100%). Error bars indicate standard error of 1. *p < 0.05.

Before HD θ-tACS, we assessed the RMT and N20 (RMT: 35.40 ± 4.90%; N20: 18.63 ± 0.44 ms). Figure 1A shows the MEPs from a representative participant before and after HD θ-tACS. The unconditioned MEP amplitude did not change between pre- and post-intervention measurements (1.41 ± 1.17 mV and 0.92 (0.45, 2.43) mV, respectively; *p* = 0.96). The conditioned MEP amplitude was slightly higher after the intervention than before the intervention (pre-HD θ-tACS: 0.46 ± 0.38 mV; post-HD θ-tACS: 0.48 (0.25, 0.69) mV; *p* = 0.093). An increase in SAI indicates less inhibition (Di Lazzaro et al., 2005b). In general, SAI decreased significantly after HD θ-tACS compared to measurements taken before the intervention (pre-HD θ-tACS: 34.90 ± 17.78%; post-HD θ-tACS: 47.80 ± 20.73%; *p* = 0.004) (Figure 1B). On an individual basis, SAI decreased in 8 out of 10 participants.

### 3.2. Effects of HD θ-tACS on the results of NDA of EEG

When subjects were in the resting state with their eyes closed, there was no significant difference between pre- and post-treatment ApEn or C-ApEn (*p* > 0.05) (Table 1). In the eyes-open state, there was no significant difference between pre- and post-treatment ApEn (*p* > 0.05), but the C-ApEn data indicated that the C3–C4 and P3–P4 connections showed a significant downward trend after the intervention (*p* < 0.05) (Table 2).

**Table 1:** ApEn and C-ApEn indices under eyes-closed conditions.

	Montages	Pre-treatment	Post-treatment	*p*
ApEn	FP1	0.73 ± 0.13	0.72 ± 0.14	0.853
	FP2	0.77 ± 0.13	0.76 (0.72, 0.81)	0.672
	F3	0.81 ± 0.13	0.82 ± 0.19	0.877
	F4	0.83 ± 0.15	0.83 ± 0.12	0.948
	C3	0.85 ± 0.13	0.82 ± 0.17	0.645
	C4	0.85 ± 0.10	0.83 ± 0.14	0.511
	P3	0.80 ± 0.11	0.79 ± 0.15	0.753
	P4	0.78 ± 0.11	0.78 (0.74, 0.83)	0.313
	O1	0.84 ± 0.11	0.80 ± 0.15	0.441
	O2	0.81 ± 0.13	0.81 ± 0.09	1.000
	F7	0.89 ± 0.10	0.88 ± 0.19	0.794
	F8	0.92 ± 0.17	0.93 ± 0.13	0.915
	T3	0.87 ± 0.15	0.84 ± 0.17	0.677
	T4	0.88 ± 0.20	0.90 ± 0.13	0.660
	T5	0.89 ± 0.16	0.83 ± 0.18	0.368
	T6	0.81 ± 0.17	0.84 ± 0.14	0.445
C-ApEn	FP1–FP2	0.97 ± 0.13	1.03 ± 0.10	0.198
	F3–F4	1.00 ± 0.11	1.05 ± 0.08	0.343
	C3–C4	1.01 ± 0.12	0.99 ± 0.08	0.519
	P3–P4	1.04 ± 0.09	1.02 ± 0.06	0.523
	O1–O2	1.05 ± 0.11	1.01 ± 0.10	0.326
	F7–F8	1.09 ± 0.10	1.08 ± 0.12	0.845
	T3–T4	1.06 ± 0.12	1.04 ± 0.13	0.467
	T5–T6	1.05 ± 0.11	1.01 ± 0.10	0.326

**Table 2:** ApEn and C-ApEn indices under eyes-open conditions.

	Montages	Pre-treatment	Post-treatment	*p*
ApEn	FP1	0.81 ± 0.14	0.75 ± 0.17	0.500
	FP2	0.84 ± 0.13	0.85 ± 0.13	0.766
	F3	0.85 ± 0.12	0.86 (0.77, 0.92)	0.541
	F4	0.86 ± 0.17	0.88 ± 0.15	0.682
	C3	0.87 ± 0.13	0.82 (0.80, 0.89)	0.594
	C4	0.88 ± 0.12	0.85 ± 0.17	0.470
	P3	0.80 ± 0.11	0.80 ± 0.13	0.960
	P4	0.83 ± 0.13	0.84 ± 0.10	0.585
	O1	0.85 ± 0.13	0.87 (0.81, 0.91)	0.683
	O2	0.84 ± 0.13	0.85 ± 0.10	0.412
	F7	0.93 ± 0.13	0.89 ± 0.18	0.434
	F8	0.96 ± 0.16	0.99 ± 0.15	0.411
	T3	0.92 ± 0.15	0.83 ± 0.16	0.147
	T4	0.90 ± 0.17	0.95 ± 0.14	0.273
	T5	0.87 ± 0.19	0.87 (0.75, 0.90)	0.477
	T6	0.83 ± 0.19	0.87 ± 0.09	0.432
C-ApEn	FP1–FP2	1.06 ± 0.11	1.06 ± 0.13	0.958
	F3–F4	1.08 ± 0.09	1.04 ± 0.08	0.315
	C3–C4	1.05 ± 0.11	0.93 ± 0.09	0.034*
	P3–P4	1.07 ± 0.10	0.98 ± 0.08	0.018*
	O1–O2	1.06 ± 0.12	0.97 ± 0.10	0.101
	F7–F8	1.13 ± 0.09	1.14 ± 0.09	0.469
	T3–T4	1.11 ± 0.08	1.06 ± 0.11	0.088
	T5–T6	1.06 ± 0.12	0.97 ± 0.10	0.101

Normally distributed continuous variables are presented in the form mean ± SD; non-normally distributed variables are presented in the form median (P25, P75). *p < 0.05.

During performance of the task, there was a statistically significant increase in ApEn at F3 after the treatment (*p* < 0.05), while in the C-ApEn data, the C3–C4, P3–P4, O1–O2, and T5–T6 connections indicated a statistically significant downward trend after HD θ-tACS (*p* < 0.05), particularly in the case of the C3–C4 connection (*p* < 0.01) (Table 3).

**Table 3:** ApEn and C-ApEn indices under memory task conditions.

	Montages	Pre-treatment	Post-treatment	*p*
ApEn	FP1	0.87 ± 0.18	0.84 ± 0.17	0.614
	FP2	0.90 ± 0.16	0.87 ± 0.12	0.285
	F3	0.86 ± 0.15	0.92 ± 0.14	0.049*
	F4	0.95 ± 0.16	0.86 ± 0.12	0.077
	C3	0.95 ± 0.15	0.92 ± 0.15	0.695
	C4	0.95 ± 0.12	0.86 ± 0.13	0.072
	P3	0.88 ± 0.16	0.93 ± 0.14	0.147
	P4	0.94 ± 0.13	0.88 ± 0.11	0.207
	O1	0.95 ± 0.14	0.94 ± 0.13	0.831
	O2	0.96 ± 0.11	0.95 (0.75, 1.02)	0.138
	F7	0.94 ± 0.14	0.95 ± 0.17	0.683
	F8	1.07 (0.93, 1.09)	1.00 (0.90, 1.05)	0.203
	T3	0.97 ± 0.16	0.96 ± 0.18	0.622
	T4	1.08 (0.82, 1.11)	0.96 ± 0.17	0.333
	T5	1.00 ± 0.17	0.98 ± 0.14	0.421
	T6	0.93 ± 0.19	0.92 ± 0.18	0.857
C-ApEn	FP1-FP2	1.14 (0.91, 1.18)	1.05 ± 0.12	0.386
	F3-F4	1.08 ± 0.14	1.03 ± 0.08	0.284
	C3-C4	1.10 ± 0.13	0.93 ± 0.10	0.007*
	P3-P4	1.12 ± 0.06	1.01 ± 0.11	0.029*
	O1-O2	1.13 ± 0.09	1.05 ± 0.13	0.049*
	F7-F8	1.13 ± 0.13	1.13 ± 0.10	0.950
	T3-T4	1.15 ± 0.10	1.10 ± 0.13	0.125
	T5-T6	1.13 ± 0.10	1.05 ± 0.13	0.049*

Normally distributed continuous variables are presented in the form mean ± SD; non-normally distributed variables are presented in the form median (P25, P75). *p < 0.05.

### 3.3. Safety and tolerability

We asked the 10 participants about their experience of receiving HD-tACS. They stated that they only experienced a slight pricking sensation when receiving the stimulation, with no other discomfort. After the experiment, we examined the intervention sites of the 10 participants and did not observe any side effects related to tACS. Furthermore, all participants exhibited good tolerance to HD-tACS. A previous study has shown that tACS may cause phosphenes or “light-flickering,” which is a characteristic side effect of tACS (Sadeghihassanabadi et al., 2022). Therefore, after the experiment, we asked participants about similar experiences, and found that none of the participants had experienced either of these symptoms.

## 4. Discussion

In previous studies, SAI has been shown to decrease with muscarinic receptor blockade (Di Lazzaro et al., 2000). Thus, SAI changes may account for the function of the cholinergic pathway by measuring sensory–motor interaction. Our preliminary research used HD θ-tACS over the left parietal cortex and observed a decrease in SAI. This drop in SAI reflects altered cholinergic transmission and cortical excitability between the parietal and motor cortices after HD θ-tACS. The results of NDA of the EEG data suggested that, compared to the resting state, interventions targeting the left parietal lobe can lead to changes in the entire frontoparietal network during task execution. This may be related to the fact that the left brain, the site of intervention, is more likely to be activated during these tasks, and the activity of the left brain also affects the interconnections between the left and right hemispheres.

There are anatomical and functional connections between the PPC and M1 (Babb et al., 1984; Chao et al., 2015). PPC acts as a relay station between the sensory and motor cortices, receiving sensory input from the sensory cortex (Chao et al., 2015). Local stimulation of the left PPC (P3), such as with TMS and transcranial direct current stimulation, has been shown to induce neuroplastic changes in M1 and to affect motor learning performance (Koch et al., 2007; Rivera-Urbina et al., 2015, 2022). In addition, θ-tACS can induce cortical θ synchronization, thereby coordinating the brain activity of sensory and motor areas to promote sensorimotor integration (Caplan et al., 2003; Cruikshank et al., 2012). Therefore, HD θ-tACS focusing on the left PPC (P3) may evoke synchronization of θ oscillation and influence the processing of incoming information from the sensory cortex through the PPC, leading to reduced inhibition and increased cortical excitability within M1, ultimately re-coordinating the activity between the sensory and motor regions.

NDA of the EEG data showed no significant differences in the connectivity of the individual brain regions when the participants were in a quiet state. In the quiet-with-eyes-open state and in the task condition, there were some changes in connectivity in certain brain regions, with the C3–C4 and P3–P4 connections in particular showing a decreasing trend. However, after receiving the intervention, F3 showed significant activation during task execution, indicating that the frontoparietal network was affected accordingly. We hypothesize that functional connectivity within the frontoparietal network decreases due to a decrease in demand for the right hemisphere as a result of interventions targeting the left hemisphere, which enhances left-brain function. Analysis of ApEn in various brain regions suggested that there was no significant change in the brain regions analyzed under the quiet-with-eyes-open condition. However, in the case of the task condition, beyond the significant increase in F3 activation, the activation of C3 and P3 also showed an increasing trend, while the activation of C4 and P4 on the non-intervention side showed a decreasing trend. These results are consistent with the findings related to C-ApEn, which demonstrate a decrease in connectivity between the left and right hemispheres. It has been demonstrated that memory retrieval, especially retrieval of episodic memory, causes left-lateralization in a large number of brain regions (Chen et al., 2017; Xue, 2018). The numerical memory task performed in this study was similar to episodic memory-related tasks; therefore, the left lateralization observed in this study is consistent with the results of previous studies. However, there no significant differences between the left and right hemispheres have been observed in other studies. Experiencing emotional interference due to the memory content during memory encoding or retrieval has also been found to lead to lateralization (Zhang et al., 2021). Despite this possibility, most studies have focused on detecting brain activity during performance of memory tasks without interventions. In this study, HD θ-tACS was administered over the left hemisphere, resulting in the possibility of easier activation of the left hemisphere during the task and thus leading to left-lateralization.

In addition, there were some differences in the frontoparietal networks before and after HD θ-tACS in both the quiet-with-eyes-open condition and the task condition. In the case of the quiet-with-eyes-open condition, the inconspicuous changes in brain regions such as P3 and C3 may be associated with participants being in a “empty” state. Under the task condition, when the participants engaged in a memory task, the relevant ipsilateral brain regions, such as F3 and C3, were in a more activated state when the P3 was reinforced by HD θ-tACS. Once the left hemisphere was more activated, the right hemisphere did not need to be “assisted.” Compared with patients with occipital lobe injury, those with frontal lobe injury show impaired performance in forward and backward digit-span tasks, indicating that the frontal lobe may be involved in the processing of explicit memory (Gong et al., 2015). The memory task in this study was related to memory for digits. Interventions were administered to the parietal lobe, so that the entire left frontoparietal network (which is associated with the digit-span type of memory task) was in a more excited state, while the occipital and temporal lobes (which are auxiliary brain regions in the memory task) showed reduced demand in the task, resulting in a decreasing trend in these two brain regions.

We analyzed data from three conditions (quiet with eyes closed, quiet with eyes open, and during the task), and found that opening the eyes had an effect on the data. Although the participants were asked to adopt a state of relaxation in the quiet-with-eyes-open condition, changes in connectivity between brain regions associated with the intervention emerged. This alteration in connectivity was more pronounced when the subjects were in the task condition. Moreover, we found that, for healthy individuals, even with the intervention, NDA of the EEG data for individual brain regions did not indicate significant change when the task was not being performed. Once the task began, there were corresponding changes in the task-related brain regions, although some of these changes were not statistically significant. This is in line with some previous findings, which have demonstrated that the activation of the frontoparietal network and the interaction between the frontoparietal network and the hippocampus during performance of such a task are conducive to memory formation and help with memory encoding (Geng et al., 2022).

It has also been shown that, in addition to memory formation and encoding, both the PPC and the hippocampus are involved in the process of memory consolidation (Zanatta et al., 1997). Although the hippocampus plays a greater role in the initial encoding and rapid learning of new information, its role gradually diminishes during learning. Conversely, the PPC will continue to function during subsequent memory consolidation and to be involved in memory representation. Consequently, greater PPC activation is associated with better memory performance (Brodt et al., 2016, 2018). In addition, rTMS over the PPC has been found to increase functional connectivity between the hippocampus and cortical regions, thus increasing memory performance (Wang et al., 2014). It can be observed that the PPC, a memory-related intervention target, can promote memory performance both through its own activity and through its interaction with the hippocampus. In this study, HD-θ-tACS induced an increase in ApEn in the left PPC (P3), suggesting that an increase in PPC activity strengthens PPC function, thus indicating the possibility of increasing functional connectivity between the hippocampus and cortical regions to improve memory coding and consolidation.

In this study, the decrease in SAI observed in participants after the intervention was administered to the PPC suggested a reduction in acetylcholine delivery. It has been shown that acetylcholine levels are important for memory encoding and consolidation, but acetylcholine modulation is selective for the role of memory function and the stage of memory processes (Gais and Born, 2004; Beer et al., 2013; Pa et al., 2013). As cholinergic activity is related to memory function, an increase or decrease in its activity is related to basal cholinergic activity. High acetylcholine levels favor memory encoding, whereas low acetylcholine levels favor memory consolidation and retrieval (Kukolja et al., 2009; Haam and Yakel, 2017). Thus, we can assume that our participants were healthy, with a high baseline performance and no problems with memory encoding. The decrease in SAI may imply a decrease in the cholinergic transmission, which is more conducive to memory consolidation in healthy participants.

## 5. Limitations

We observed changes in brain function after the intervention was administered to healthy participants, as evidenced by the results on SAI and NDA of the EEG. Unlike in other studies, we did not perform reinforcement of the memory task during the intervention, so our results only suggest the influence of the HD θ-tACS intervention in the left PPC. In addition, the correct or incorrect nature of participants’ responses was not determined as part of the memory task in this study. Thus, we observed only the invocation of the brain network during the memory task and the changes in activity in each region. Since NDA of EEG data can only account for the cortical regions, rather than the subcortical regions, our findings cannot determine the connectivity between the hippocampus and PPC after the intervention. Therefore, future studies should use imaging methods that can visualize the subcortical regions to conduct a more comprehensive analysis of the changes in brain function induced by HD θ-tACS as a result of its action on the PPC, such as the connectivity between the PPC and hippocampus at various stages of memory processing. This will provide additional evidence regarding the effects of tACS on memory function and the mechanisms underlying the action of tACS on the parietal cortex.

## 6. Conclusion

HD theta-tACS is a potential treatment that can influence the cholinergic circuits, strengthen the frontoparietal network by acting on the left parietal cortex, and cause left-lateralization, which may facilitate memory formation, encoding, and consolidation. These findings may help in designing future interventions for patients with memory impairment. If the deeper brain networks can be explored using other imaging methods, better assessment and treatment targets can be identified for clinical purposes.

## Data availability statement

The original contributions presented in the study are included in the article/supplementary material, further inquiries can be directed to the corresponding authors.

## Ethics statement

The studies involving humans were approved by Ethics Committee of Yueyang Integrated Hospital of Traditional Chinese and Western Medicine Affiliated to Shanghai University of Traditional Chinese Medicine. The studies were conducted in accordance with the local legislation and institutional requirements. The participants provided their written informed consent to participate in this study.

## Author contributions

XC: Writing – original draft. YW: Writing – original draft. XS: Investigation. ZZ: Investigation. TF: Investigation. MR: Writing – review & editing. YL: Conceptualization. CS: Conceptualization.

## Footnotes

^1^https://www.chictr.org.cn/index.aspx
