# Erratum: Neuromodulatory effects of high-definition theta transcranial alternating current stimulation on the parietal cortex: a pilot study of healthy males

**DOI:** 10.3389/fnins.2024.1471095

**Published:** 2024-08-06

**Authors:** Frontiers Production Office

**Affiliations:** Frontiers Media SA, Lausanne, Switzerland

**Keywords:** transcranial alternating current stimulation, parietal cortex, short latency afferent inhibition, EEG, Nonlinear dynamics analysis

Due to a production error, the publication date in the PDF version of the article was incorrect.

A correction has been made to the publication date in the PDF version of the article:

“PUBLISHED 09 November 2023”

The publisher apologizes for this mistake. The original article has been updated.

